# Chemical named entity recognition in the texts of scientific publications using the naïve Bayes classifier approach

**DOI:** 10.1186/s13321-022-00633-4

**Published:** 2022-08-13

**Authors:** O. A. Tarasova, A. V. Rudik, N. Yu. Biziukova, D. A. Filimonov, V. V. Poroikov

**Affiliations:** Laboratory of Structure-Function Based Drug Design, Institute of Biomedical Chemistry, 10 bldg. 8, Pogodinskaya Str., Moscow, 119121 Russia

**Keywords:** Chemical named entity recognition, CNE, CNER, Naïve Bayes classifier, SARS-CoV-2, Mpro inhibitors

## Abstract

**Motivation:**

Application of chemical named entity recognition (CNER) algorithms allows retrieval of information from texts about chemical compound identifiers and creates associations with physical–chemical properties and biological activities. Scientific texts represent low-formalized sources of information. Most methods aimed at CNER are based on machine learning approaches, including conditional random fields and deep neural networks. In general, most machine learning approaches require either vector or sparse word representation of texts. Chemical named entities (CNEs) constitute only a small fraction of the whole text, and the datasets used for training are highly imbalanced.

**Methods and results:**

We propose a new method for extracting CNEs from texts based on the naïve Bayes classifier combined with specially developed filters. In contrast to the earlier developed CNER methods, our approach uses the representation of the data as a set of fragments of text (FoTs) with the subsequent preparati`on of a set of multi-*n*-grams (sequences from one to *n* symbols) for each FoT. Our approach may provide the recognition of novel CNEs. For CHEMDNER corpus, the values of the sensitivity (recall) was 0.95, precision was 0.74, specificity was 0.88, and balanced accuracy was 0.92 based on five-fold cross validation. We applied the developed algorithm to the extracted CNEs of potential Severe acute respiratory syndrome coronavirus 2 (SARS-CoV-2) main protease (Mpro) inhibitors. A set of CNEs corresponding to the chemical substances evaluated in the biochemical assays used for the discovery of Mpro inhibitors was retrieved. Manual analysis of the appropriate texts showed that CNEs of potential SARS-CoV-2 Mpro inhibitors were successfully identified by our method.

**Conclusion:**

The obtained results show that the proposed method can be used for filtering out words that are not related to CNEs; therefore, it can be successfully applied to the extraction of CNEs for the purposes of cheminformatics and medicinal chemistry.

**Supplementary Information:**

The online version contains supplementary material available at 10.1186/s13321-022-00633-4.

## Introduction

An analysis of texts is essential for extracting new knowledge about chemical compounds, drugs, targets, pathological processes and diseases; it allows determining various relationships including identification of molecular mechanisms, pharmacological effects and toxicity of drug, pathophysiological processes and determining drug-target-disease relationships [1, 2]. Extraction of chemical named entities (CNEs) from scientific publications is an essential task since it allows using the obtained data for building chemical-target associations [3], leading to improvement of the data curation [3–6]. Chemical named entity recognition (CNER) algorithms can help create large sets of named entities of chemical compounds associated with physical and chemical properties or biological activities [3, 4]. Therefore, such algorithms allow the identification of CNEs and the collection of big data on chemicals and their properties [7–15]. Both dictionary-based and machine learning approaches used to reveal CNEs have limitations due to the peculiarities of text structure. Since scientific publications are low-formalized sources of information and CNEs make up only a small part of the whole text, the datasets used for training are highly imbalanced. The extraction of CNEs is restricted by the incompleteness of the representation of chemical names and properties of chemical compounds in the texts [7]. For instance, they might be provided for the scaffolds but not for whole chemical compounds. Another restriction is associated with the variability of the chemical names, where various punctuation marks can be found, including dots, commas, hyphens, etc. In this case, the accuracy of the named entity recognition approach would be very sensitive to the specific method of tokenization, i.e., breaking up the input lines into pieces such as keywords, phrases, symbols, etc. [7]. The information about parameters of chemical and physical properties of chemical compounds also lack reproducibility [3, 4].

An obvious disadvantage of the rule-based and dictionary-based methods is a limited number of CNEs that can be recognized due to the fixed size of dictionaries or rule numbers. Machine learning or artificial intelligence approaches [16, 17] mainly use support vector machines [18] or artificial neural networks [8, 14] including deep learning architectures [19]. Typically, these methods can reach an accuracy of approximately 85–95% [8, 14, 17, 18]. Some methods are sensitive to imbalanced data [20]. Long-short term memory networks (LSTM) [12–14, 21–24] or conditional random fields (CRF) [16, 25] are efficiently applied to the task of named entity extraction. The architecture of neural networks can be modified according to the particular task of CNER [26–28].

Recently, BERT (Bidirectional Encoder Representations from Transformers) and derived approaches have demonstrated high performance in several named entity recognition tasks, especially in the domain-specific area of materials science [29]. Big Data (a pre-training corpus containing about 8.8 billion tokens) and supercomputer (the Bridges-2 supercomputer at the Pittsburgh Supercomputing Center) were used to achieve high performance of recognition [29].

Computational machine learning approaches require text preprocessing followed by the generation of vector-based or sparse word text representation (one-hot encoding). The accuracy of CNER, to some extent, is dependent on the completeness of the corpus that was used to create the set of vectors and its relevance to a particular task (i.e., chemical named recognition, biological named recognition, the search of chemical-biological associations, etc.). There is a constant need in development of approaches providing possibility of their use by many researchers in the bridging field of chemoinformatics including medicinal chemistry, computational biology, drug discovery, material science, etc. The new methods aimed at easy-to-use, accurate and fast CNER are still in demand [16].

Our study represents the new approach for CNER in the texts of scientific publications based on the naïve Bayes classifier (NBC). We previously evaluated the applicability of an in-house developed NBC for predicting the biological activities of chemical compounds and estimating viral drug resistance [30]. Originally developed multilevel neighbourhoods of atoms (MNA) descriptors were used for the representation of chemical structures. However, further development of the method allows us to use short peptide and nucleotide sequences (represented by a sequence of symbols) in our algorithm [30]. These results demonstrated the applicability of the NBC approach to the classification of sequences according to a certain task. We demonstrated that our approach can be applied for prediction even in the case of highly imbalanced datasets [30, 31].

A newly developed approach based on NBC is aimed at the recognition of CNEs and their classification into types (trivial, systematic, etc.) in the abstracts of scientific publications based on the set of n-grams of length from one to n symbols (the so-called multi-n-grams), generated for each symbol of a text. We have built an application that can perform colouring of the text fragments that were recognized as belonging to CNEs and various types. We evaluated the accuracy of CNER on the large, labelled corpus of CNEs freely available via the internet (CNEMDNER). Additionally, we tested our method in an application for CNER of potential anti-SARS-CoV-2 agents. We used a collection of texts relevant to SARS-CoV-2 main protease (Mpro) inhibitors. CNEs were extracted from the texts, and the accuracy of extraction was verified using automated and manual analysis. Based on the CNE extraction procedure, we were able to identify a set of entities related to the chemical compounds assayed against SARS-CoV-2 Mpro inhibition, and for some of them, inhibitory activity against Mpro was confirmed.

## Material and methods

### Text corpus

To perform CNER, we used the freely available corpus CHEMDNER [17]. CHEMDNER contains 10,000 abstracts. CNEs are labelled by an expert for each abstract. CHEMDNER consists of over 80,000 labelled named entities of chemical compounds for over 19,000 unique named entities [17]. CHEMDNER contains a set of texts compiled based on NCBI PubMed [32] abstracts, in which CNEs are represented by the position of the first and the last symbol in the text. In our study, we used annotation for the labelled texts that assigns the chemical named entities to one of the following types: “Abbreviation” (mainly abbreviations and acronyms), “Systematic” (IUPAC names of chemicals), “Formula” (the formula in the text that is associated with the CNE), “Family” (family of chemicals), and “Trivial” (trivial names of chemicals and trademark names) [17]. The “non-CNE” type was not arranged to any CNE and represented any other fragment of text (FoT). Additionally, we merged all CNEs belonging to different types into one, chemical named entity (“CNE”). Examples of the CNEs of each type are provided in Table 1.

**Table 1:** Examples of chemical named entities belonging to particular types

Type	An example of A chemical named entity
Abbreviation	Mtx
Systematic	Anthracene, phenylenediamine
Formula	H_2_O_2_
FAMILY	Flavonoids
Trivial	Haloperidol
Chemical Named Entity (CNE)	﻿Anthracene, phenylenediamine, haloperidol, H_2_O_2_, Mtx, flavonoids
Non-CNE	Quarantine

**Fig. 1:**
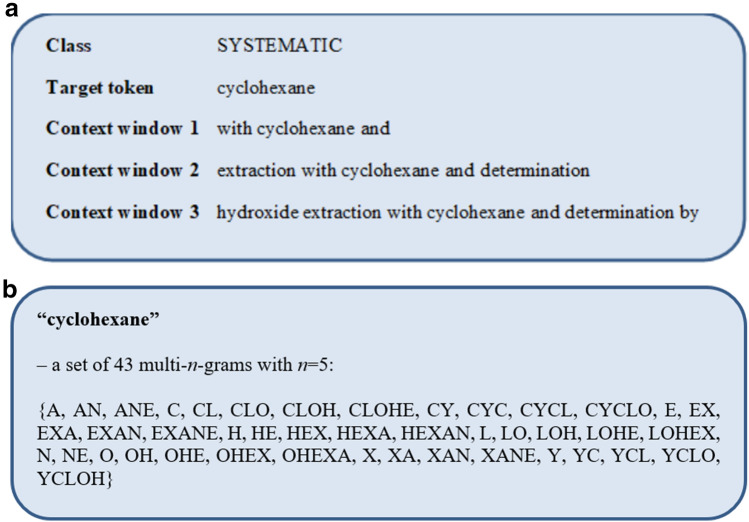
**a** The types are arranged for each variant: target token and a set of tokens before and after the target token; **b** an example of a set of multi-*n*-grams with *n* = 5 generations

CHEMDNER was tokenized. We used the Python NLTK WordPunkt tokenizer [33]. All the types denoted above were arranged into four FoT variants of FoT by the context window: the central token (the so-called target token) and the FoTs obtained by concatenating one, two, or three tokens before and after the target token (see Fig. 1a). The set of FoTs prepared for CHEMDNER is available in the Supplementary Materials in SDF format (Additional file 1). We built the set of descriptors by shifting target tokens one by one. If the obtained FoT does not belong to any type, it is assigned to the "non-CNE" type [17]. Then, for each set of tokens belonging to a particular type, we generated a set of *n-*grams (the so-called multi-n-grams), which are continuous sequences of one to *n* symbols from a token. We used five types of *n-*grams with *n* values from one to five (see Fig. 1b).

### The algorithm of named entity recognition

The CNER algorithm is based on the NBC approach implemented in the Prediction of Activity Spectra for Substances (PASS) software [34]. The standard usual NBC is a linear classifier:

$${\varvec{if}} \, {\varvec{a}}{\boldsymbol{^{\prime}}}{\varvec{x}} \, > \, {\varvec{b}} \, {\varvec{then}} \, Class 1 \, {\varvec{else}} \, Class 2$$

where ***a*** are the coefficients, *x*_*i*_ = 1 or *x*_*i*_ = 0 for the *i*-th feature (descriptor) of the classified object, and *b* is the threshold value. Unlike linear regression or any other similar methods, the NBC coefficients are not the result of optimization, but they are calculated directly based on the training dataset. According to the naïve Bayes approach, the a posteriori probability $P\left(C|F\right)$ that the FoT $F$, which is represented by the set $\{{\mathrm{g}}_{1},\dots ,{\mathrm{g}}_{m}\}$ of *m* descriptors, the *n*-grams ${\mathrm{g}}_{i}$, belongs to type *C* satisfies the equation:

$$ln\left[\frac{P\left(C|F\right)}{1-P\left(C|F\right)}\right]\cong ln\left[\frac{P\left(C\right)}{1-P\left(C\right)}\right]+{\sum }_{i=1}^{m}\left\{ln\left[\frac{P\left(C|{\mathrm{g}}_{i}\right)}{1-P\left(C|{\mathrm{g}}_{i}\right)}\right]-ln\left[\frac{P\left(C\right)}{1-P\left(C\right)}\right]\right\},$$

where $P\left(C\right)$ is the a priori probability of type $C$ and $P\left(C|{\mathrm{g}}_{i}\right)$ is the conditional probability of type $C$ for a particular descriptor, *n*-gram ${\mathrm{g}}_{i}$.

The explanation of this expression is quite clear: the logarithm of the a posteriori likelihood ratio is the sum of the logarithm of the a priori likelihood ratio and the sum of individual descriptor contributions. If the type of FoTs is not dependent on the *n*-gram ${\mathrm{g}}_{i}$, then $P\left(C|{\mathrm{g}}_{i}\right)=P\left(C\right)$, and the contribution of the *n*-gram ${\mathrm{g}}_{i}$ to the sum is zero. The expression in the curly brackets is the value of coefficient *a*_*i*_ in the standard NBC.

However, this simple result of the usual naïve Bayes approach has a significant well-known disadvantage: the contribution of some descriptors, for which the conditional probability of activity is too close to 0 or 1, is too large and suppresses all other terms of the sum. The PASS algorithm uses the so-called arcsine Fischer transform instead of $ln[p/(1-p)]$. The shape of $ArcSin\left(2p-1\right)$ coincides with the shape of $ln[p/(1-p)]$ for almost all values of p, but the $ArcSin\left(2p-1\right)$ values are bounded by the values ± π/2. The accuracy of the PASS prediction also improved after changing the sum by the average value. The a priori likelihood ratio is constant, does not contain information about a specific recognized FoT, and can be omitted.

The naïve-Bayes CNER algorithm is based on the specific *B*-statistics, which are calculated according to the following expressions:

$$P\left({C}_{k}\right)= \frac{{N}_{k}}{N}, P\left({C}_{k}|{\mathrm{g}}_{i}\right)= \frac{{N}_{ik}}{{N}_{i}},$$

$${S}_{0k}=2P\left({C}_{k}\right)-1, {S}_{k}=Sin\left[{\sum }_{i=1}^{m}ArcSin\left(2\left({C}_{k}|{\mathrm{g}}_{i}\right)-1\right)\right],$$

$${B}_{k}=\frac{{S}_{k}-{S}_{0k}}{1-{S}_{k}\cdot {S}_{0k}},$$

where $N$ is the number of FoTs (tokens) in the training set and ${N}_{k}$ is the number of FoTs belonging to the type ${C}_{k}$.

We evaluate the recognition accuracy based on the leave-one-out cross-validation (LOO CV) and fivefold cross-validation (5-F CV) procedures using the invariant accuracy (IA) estimates [34]. IA reflects the probability that the estimate *E*(*FoT*) assigns the higher value to a randomly selected positive example *FoT*_+_ than to the randomly selected negative example *FoT*_-_. The IA coincides with the AUC (popular in ML the Area Under the ROC Curve), but has a simpler and clearer definition:

$$IA=\frac{\#\left[E\left({FoT}_{+}\right)>E\left({FoT}_{-}\right)\right]}{\#[{FoT}_{+}]\cdot \#[{FoT}_{-}]}$$

where #[*x*] is the number of cases *x*. IA is calculated for all pairs of positive and negative examples in the validation dataset.

During the execution of the LOO CV procedure, polynomial estimates of the distributions of *B*-statistics for the CNE and non-CNE classes are calculated, and on this basis, estimates of the probability *P*_*c*_ of belonging and *P*_*nc*_ of not belonging to CNE are constructed as functions of *B*-statistics.

CNER was performed based on the values of *P*_*c*_ and *P*_*nc*_ after determining the threshold (for details, please see the “Results and Discussion” section).

Automated validation of CNER was performed using a set of scripts (Python 3.7.4) that were built to perform automated queries to the PubChem and ChEMBL databases. The scripts are provided in the Supplementary Materials (Additional file 2). The values of accuracy are provided in the “Results and Discussion” section.

The developed approach was tested on a CNER task using the CHEMDNER corpus and in retrieving CNEs of potential SARS-CoV-2 inhibitors. The values of balanced accuracy and F1-score for CHEMDNER were evaluated using 5-F CV. To test our approach on the task of CNER for potential anti-SARS-CoV-2 agents, we created a test set as described below.

The text collection *SARS-CoV-2 Mpro set* was compiled based on the text query that included all synonyms of SARS-CoV-2 main protease (Mpro) and the term “inhibitors”. The synonyms of Mpro were retrieved from UniProt [35]. The full text of the query and a set of texts are provided in the Supplementary Materials (Additional file 2). In total, we collected 1,528 texts. The texts were processed using Python NLTK WordPunkt tokenizer yielding 484,676 tokens. We used the model built on multi-*n*-grams with a maximum of five symbols and a context window of one, as it was characterized by the highest accuracy values. The performance of the model was estimated semiautomatically using an iterative multistage procedure.

## Results and discussion

**Fig. 2:**
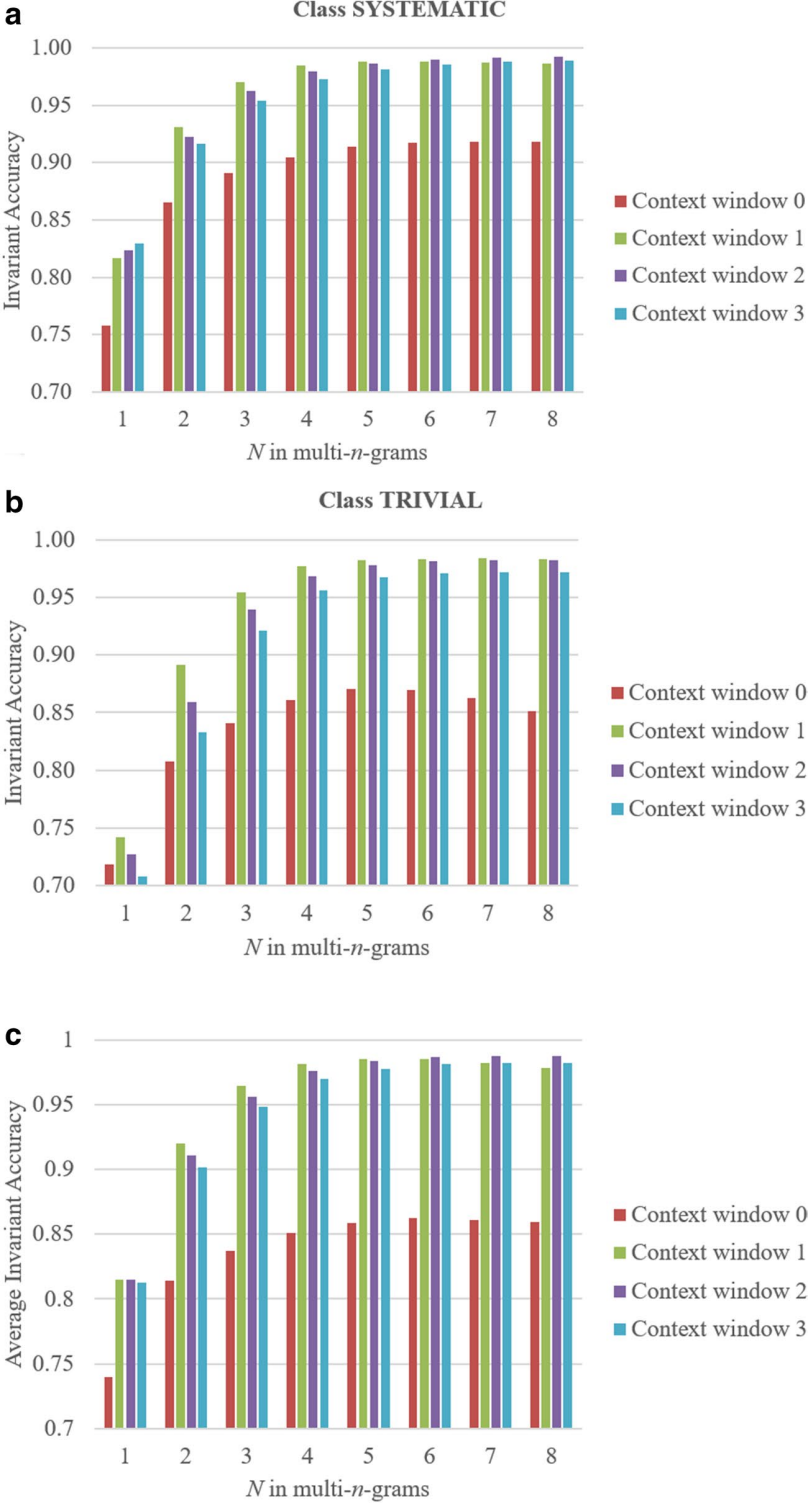
The relationship between length of an n-gram, context window, and accuracy of CNER: **a** for class “Systematic”, **b** for class “Trivial”, and **c** average IA for all classes

We evaluated the most suitable parameters for high accuracy of CNER using NBC based on IA values. The highest IA values were achieved with context windows one and two, i.e., one (two) token before and one (two) token after the target token (see Fig. 2) depending on the class. For the systematic class, the highest IA values are obtained for context window one, multi-*n*-grams with *n* = 5 (IA = 0.988), and context window two, multi-*n*-grams with *n* = 7 (IA = 0.992), and the difference in accuracy values for these parameters is insignificant. For the class Trivial, the highest IA value was achieved for the following parameters: context window one, multi-n-grams with *n* = *7* (IA = 0.984). Figure 2 shows that the values of IA increase along with an increment of the maximal context window up to two tokens before and two after a target token for the class Systematic; however, this is not the case for the class Trivial, for which the highest IA values were obtained for context one. The highest average IA values can be achieved for the following parameters: context window one and multi-n-grams with *n* = *5* (IA = 0.986); almost similar values of IA can be obtained with context window two and *n* = *7* (IA = 0.988). Therefore, the IA values do not grow dramatically with an increase in the context window of more than one token and multi-n-grams of more than five symbols. These observations allow us to conclude that a combination of a context window, one token before and after a target token, and maximum multi-n-grams of five can be used to achieve the highest accuracy of CNER based on the naïve-Bayes approach, and it provides reasonable computational complexity.

The IA values and balanced accuracy (BA) of CNER for various classes obtained in leave-one-out-cross-validation are given in Table 2. The IA, sensitivity (recall), precision, specificity, and BA values for various thresholds of *B*-statistics are provided in the Supplementary Materials (Additional file 3).

**Table 2:** Accuracy of chemical named entity recognition using the naïve-Bayes approach based on the representation of texts using n-grams equal to five symbols and a context window of one token before and after analysis

Type	N*	R**	IA*** (loo cv)
Abbreviation	12,506	118	0.99
Formula	13,466	110	0.99
Family	19,017	78	0.97
Systematic	32,510	46	0.99
Trivial	25,140	59	0.98
CNE	102,639	14	0.98
Non-CNE	1,480,509	1.01	0.98

^*^—N is the number of fragments of texts used for training

^**^—R is the ratio of the number of all tokens to the number of tokens

belonging to a certain type, indicating a measure of dataset imbalance

^***^—IA invariant accuracy

We surmise that the decrease in accuracy for *n-*grams of seven or more symbols may be associated with the high uniqueness of such long *n-*grams in the training set.

These results may also be associated with the peculiarities of the text fragment formation: the higher number of tokens arranged to the type may lead to difficulties in recognizing the features of the target token. At the same time, a minimal context of one token before and after the target token can help consider whole words or the parts of terms that can point to a chemical named entity, such as “inhibitor”, “drug”, “chemical”, and “substance”.

We should emphasize that the number of tokens of the “NON-CNE” type is approximately 50 times higher than the number of tokens of types “Systematic” and “Trivial”. The results of Table 2 show that the data imbalance does not influence the values of accuracy IA. Similar results we obtained earlier for the Bayes-based approach applied to the prediction of HIV resistance [20]. Based on the interpretation of accuracy for “CNE” and single, complex substance NEs, we can propose that CNEs can be extracted from the texts of abstracts for further analysis using the prediction results.

Utilizing naïve-Bayes approach for CNER, we investigated the relationship between recall (sensitivity), precision, specificity, and balanced accuracy for the context window of one and multi-*n-*grams with *an n* value of one to five for the CHEMDNER corpus.

**Fig. 3:**
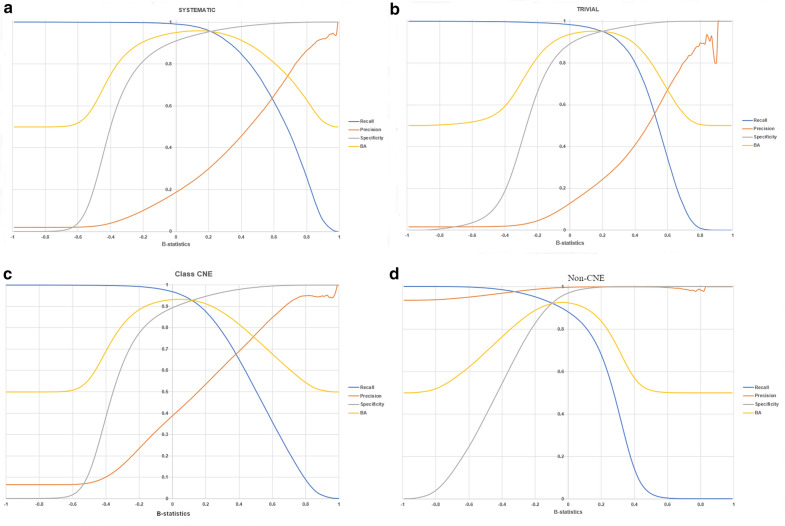
The relationships between the values of accuracy and *B-*statistics for the types: **a** “Systematic”; **b** “Trivial”; **c** “CNE”; **d** “non-CNE”

Figure 3 shows the relationships between the values of accuracy metrics (precision, recall, specificity, BA) and *B-*statistics for the “Systematic” and “Trivial” types (the most represented classes in the training set), CNE, and non-CNE type. The relationship between the accuracy metrics and *B-*statistics for all other types is provided in the Supplementary Materials (Additional file 3).

As shown in Fig. 3, the recall and BA values have similar patterns of curve growth and decline, while the patterns of recall and precision curves are different. In particular, the precision curve increases while the recall curve decreases, and vice versa. It is obvious and occurs because the number of false-positives decreases while the number of false-negatives increases. A small number of positive samples (Systematic and Trivial types in Fig. 3a, b) in the training set may explain the more flattened pattern of the precision curve. Comparison of the curve growth and decline patterns reveals that they are not related to any chemical named entity and provides the opportunity to compare accuracy metrics. For instance, for Trivial and Systematic types, the precision curve character changes significantly depending on the threshold, while for the non-CNE type, the situation is different. It allows making a conclusion that the precision is rather sensitive to the imbalance of the data, while sensitivity (recall), specificity, and balanced accuracy are not sensitive to the data imbalance. Another feature of precision is its sensitivity to the threshold choice (see Fig. 3). When a method is designed to extract information on chemical named entities from texts, the values of specificity and sensitivity (recall) are essential for validation because they help estimate the proportion of false-positives. A method with high specificity and sensitivity (recall) values provides the possibility to extract the correct chemical named entity based on the estimates of probabilities that indicate belonging to CNE and non-CNE as a consensus result.

**Fig. 4:**
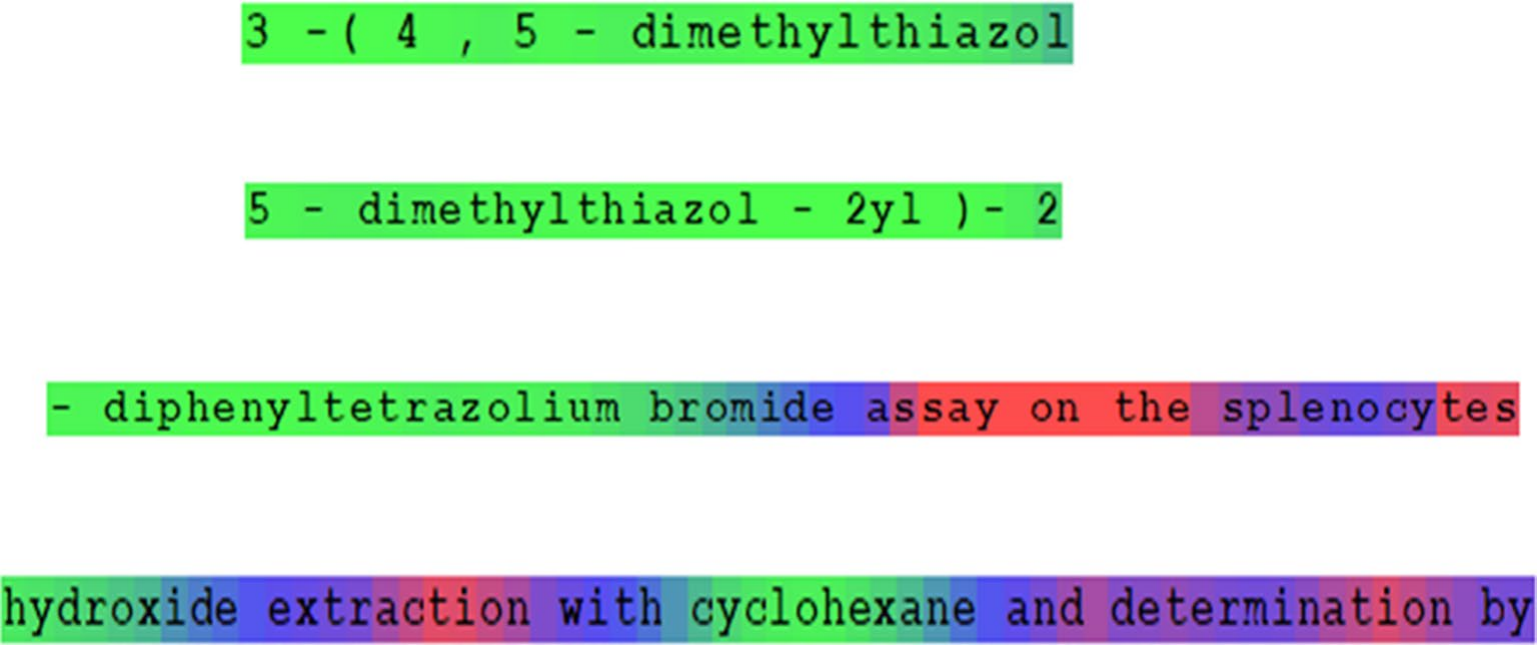
An example of chemical named entity extraction based on naïve-Bayes estimations

Our CNER algorithm also allows evaluating each symbol in an FoT. A set of *n*-grams including a particular position in the FoT is used. In Fig. 4, for the letter "o" in the token "cyclohexane", the set of *n*-grams {O, LO, OH, CLO, LOH, OHE, YCLO, CLOH, LOHE, OHEX, CYCLO, YCLOH, CLOHE, LOHEX, OHEXA} with n = 5 is used for estimation. The values *P*_*c*_ = 0.915 and *P*_*nc*_ = 0.002 are calculated for the letter "o" in the token "cyclohexane" for class "SYSTEMATIC". On such bases in our naïve-Bayes CNER approach, the colouring of FoTs is used. The colour of the letter corresponds to light green for *P*_*c*_ = 1 (*P*_*nc*_ = 0), light red for *P*_*nc*_ = 1 (*P*_*c*_ = 0), and blue when *P*_*c*_ and *P*_*nc*_ are both close to zero.

Chemical named entities can be extracted after tokenization of texts and making a prediction for each token based on the values of *P*_*c*_ and *P*_*nc*_. Extraction of a chemical named entity can be performed by concatenating the tokens predicted to belong to a CNE class.

### Validation of the naive-based approach in the task of extracting chemical named entities

#### Extracting chemical named entities based on the CHEMDNER corpus

We checked the applicability of our approach for extracting chemical named entities and tested it in a case study of CNER extraction using CHEMDNER.

To extract chemical named entities, we ought to determine the best strategy for extracting chemical named entities by a naïve Bayes-based approach.

First, we evaluated the threshold for extracting chemical named entities based on the recognition results. We calculated a set of values (*Pc-Pnc)* that corresponds to the highest accuracy of distinguishing tokens that belong and do not belong to CNE. The value of the threshold was obtained empirically. In particular, we evaluated the sensitivity (recall), precision, specificity and balanced accuracy for each threshold value using five-fold cross validation. Then, we selected the threshold of 0.3 because it was associated with an optimum combination of values of sensitivity (recall), precision, specificity and balanced accuracy.

Then, we extracted named entities as the concatenated sequence of tokens with (*P*_*c*_*-P*_*nc*_*) *above the *T value.* To improve the extraction procedure, we applied some filters aimed at exclusion of tokens that obtain high values of (*P*_*c*_*-P*_*nc*_*)* because they are overrepresented in the training set (for instance, numerical values, single brackets, etc.). In addition, the named entities with incorrect encoding were removed from the set of extracted CNEs, disregarding the prediction results. The set of filters is provided in the Supplementary Materials (Additional file 4).

The values of precision, sensitivity (recall), specificity, and balanced accuracy for CHEMDNER were evaluated using five-fold cross-validation. The files created for CHEMDNER is provided in in the Supplementary Materials (Additional file 5). For the CHEMDNER dataset, the sensitivity (recall) was 0.95, precision was 0.74, specificity was 0.88, and balanced accuracy was 0.92. These values of accuracy represent the approximate performance of recognition for the whole chemical named entities, not their parts.

#### Extracting named entities of potential anti-SARS-CoV-2 agents

Text and data mining approaches are very helpful in extracting information relevant to pathological processes in the human body, disorders, side effects of drugs, etc. Therefore, we suppose that it is important to test the applicability of our approach for solving practical tasks that may have a high clinical and biological impact of current interest. We investigated the possibility of extracting named entities of chemicals that can inhibit SARS-CoV-2 main protease (Mpro) and slow down COVID-19 progression. We chose to extract inhibitors of SARS-CoV-2/COVID-19 as a case study because of the availability of large collections of texts relevant to SARS-CoV-2 studies. We suppose that an analysis of the chemical names extracted from texts relevant to the SARS-CoV-2 Mpro inhibition can help to identify some trends in the particular chemical compounds, their classes and families that are most commonly tested against SARS-CoV-2 Mpro.

We extracted a total number of 8,071 named entities corresponding to 2,649 unique CNEs. Then, we calculated the precision value for the extracted examples using automated annotation followed by a manual inspection. First, we performed automated queries of the PubChem [36] and ChEMBL [37] databases. Such queries allowed us to estimate the number of true-positive samples automatically. Then the results of automated queries were checked. In total, 4,374 named entities were found in the databases correctly (1,201 CNEs without duplicates). For those CNEs that were not found in the databases we performed a manual annotation of extracted named entities classifying them onto CNE and non-CNE. Manual examination of retrieved CNEs allowed us to identify additionally 1,407 NEs extracted correctly (507 CNEs without duplicates). Based on the results of manual and automated validation, we calculated precision for the *SARSCoV-2 Mpro set* which was 0.72.

During a manual inspection of the entities recognized as belonging to CNEs according to naïve-Bayes CNER, we noticed that some entities were identified correctly but were not found in the PubChem and ChEMBL databases. Some of them (1%) were identified as CNE by the naïve-Bayes algorithm, but they were not found in the databases because of misprints (for instance, such named entities include "hydroxybenzoagte" (the correct name: hydroxybenzoate) and "dithiazone" (the correct name: dithizone). Another part consisting of 1% found entities were codes of chemical compounds provided in the publication and therefore had the context indicating that the entity is CNE. Approximately 6% were recognized but were not found in PubChem because they belong to chemical families. The naïve-Bayes model was based on the merged class CNE, which includes chemical families; therefore, they were recognized by naïve-Bayes approach but, naturally, were not found in PubChem. Examples of such named entities include “ginsenosides”, “flavonoids”, “triterpenoids”. Chemical named entities that are natural compounds have not been found via automated queries of the PubChem database. The names of bioactive peptides and incomplete chemical named entities as well as all other terms were regarded as false-positives. The extracted CNEs are provided in the Supplementary Materials (Additional file 4).

Manual analysis of the true-positive chemical named entity mentions allowed us to identify several names of chemical compounds that were evaluated for inhibition of SARS-CoV-2 (for instance, hydroxychloroquine, chloroquine, quercetin, rutin, curcumin, darunavir, saquinavir, and flavonoids).

Although chloroquine and hydroxychloroquine are the most thoroughly investigated drugs and therefore appeared in the set of chemical named entities extracted from the texts collected by a query associated with SARS-CoV-2 Mpro, they were considered ineffective after a set of studies [38]. Quercetin was experimentally tested for its activity against SARS-CoV-2 Mpro and demonstrated inhibitory activity [39]. Flavonoids represent a group of natural compounds (secondary plant metabolites) that are widely discussed in the scientific literature and are considered to have anti-inflammatory effects and the ability to modulate cytokines [40]. The inhibitory effect of some flavonoids (tangeretin, gardenin B) on SARS-CoV-2 was demonstrated [40]. The anti-inflammatory activity and inhibitory activity of dihydromyricetin on SARS-CoV-2 Mpro were evaluated in a FRET assay (fluorescence resonance energy transfer) [41]. It was shown that the half-maximal inhibitory concentration of SARS-CoV-2 Mpro by dihydromyricetin reached 1.76 µM. Additionally, the authors [42] confirmed the activity of dihydromyricetin on the proteins included in the TGF-β 1/Smad pathway, which are responsible for the development of pulmonary fibrosis.

These results demonstrate the applicability of the Bayes-based CNER approach to the extraction of CNEs in the text of abstracts relevant to a particular task and therefore allow the scientific community to enrich the knowledge about potential chemical compounds effective against particular targets and can be used for the treatment of specific diseases, including novel humanity threats such as COVID-19.

#### A place of the naïve-Bayes CNER among other methods

Texts of publications represent low-formalized data, and their classification may be difficult even for experts in the field. In contrast to approaches that take any semantical or grammatical features of a token, our method takes the text data as input without any additional processing into parts of speech and other grammatical or semantic features.

Many various artificial intelligence (AI) approaches aimed at chemical and biological named entity recognition have been developed [15, 18, 21]. Most approaches that have been under recent development for several years are based on the usage of neural networks with different variants of long-short term memory (LSTM) architecture or conditional random fields (CRF) [16, 42].

Many NER algorithms based on machine learning use discriminative probabilistic graphical model, a particular example of which are conditional random fields (CRF) [43]. As an input, CRF based models require a set of parameters for sequences of tokens. Our previously developed approach for CNER based on CRF allows extraction of chemical named entities with precision 0,91 and recall 0,87 [16, 42]. Tang and colleagues [44] performed a comparison of CRF-based and structured support vector machines (SSVM)-based CNER model performance. Using the same set of features, SSVM-based method demonstrated close performance comparing to CRF-based one: the values of precision were 0.88 and 0.89 and recall 0.83 and 0.81, respectively.

Some algorithms are based on deep learning methods and use neural networks (NN) with multiple layers. Common architecture in such tasks is a variety of recurrent neural networks—long-short term memory (LSTM). A modification of the LSTM with forward and back propagation of the signal is used—bidirectional LSTM (BiLSTM) is typically used for NER. LSTM architecture can be used in combination with some other techniques. In the study by I. Korvigo and co-authors [19] word- and character-level embedding was used to describe texts. While trained on CHEMDNER corpus, the model reached precision and recall 0.89 and 0.89 for CNER, respectively. In the other study, the combination of BiLSTM and CRF was used [45]. The authors provide values of precision and recall for two models: CRF-BiLSTM (CHEMDNER: precision 0*.*92, recall 0.89) and CRF-BiLSTM with attention layer (CHEMDNER: precision 0.92, recall 0.90).

Using pre-trained models, such as the mentioned above NN-based BERT, may improve the performance of NER algorithm. To increase the accuracy of recognition in biomedical text mining tasks, BioBERT was developed [46]. The authors compared the performance of their model with BERT. The precision corpus for BioBERT was 0.92 compared to 0.91 (BERT), recall 0.91 compared to 0.89 based on CHEMDNER.

Most AI-based approaches initially convert text into vectors or use sparse word text representation created with preprocessing of a text corpus, and vector preparation (for instance, such approaches include word embedding preparation or the one-hot-encoding technique). It should be noted that the performance of CNER using the naïve-Bayes approach, in general, is comparable with most of earlier published methods [16, 18, 22–25], while it is slightly lower comparing to some other approaches based on the results of fivefold CV [19, 45, 46].

The presented method is simple for application and it does not require re-training after enlargement of the corpus transformation into vectors. The latter feature provides the versatility of our method in its application to very different text styles and language peculiarities, which can also include some specific changes in the language grammar and lexical features that can occur during natural evolution of language.

It should be noted that we evaluated the accuracy of CNE extraction in addition to the accuracy of a particular token belonging to the specific class. Therefore, texts relevant to various queries can be processed efficiently using the developed naive-based approach. Extraction of novel chemical entities may be rather helpful for the purposes on novel drug design including both experimental studies and cheminformatics approaches, virtual screening that represent a group of powerful approaches for exploring large chemical space [4, 47].

## Conclusions

We developed a new naïve Bayes classifier approach for extracting chemical named entities from texts of scientific publications. The features that we used included the sequential tokens merged by three: a “target token”, one token displaced in the text before and one token after the target token. Text strings collected from merged tokens are represented as a set of multi-n-grams, consisting of one to five symbols. The algorithm provides an estimation of the probability for each token belonging to the following classes: “Abbreviation”, “Family”, “Formula”, “Systematic”, “Trivial”, “CNE” (an integrated class that includes the aforementioned classes), and “non-CNE”. Our approach allows the prediction of a token belonging to the classes with an invariant accuracy of 0.96–0.99.

The presented approach uses neither linguistic basis, nor typical handling for standard natural language processing approaches. The approach is based on the representation of text as a set on multi-n-grams, which are similar to sub-structural descriptors of molecules in (Q)SAR studies. The developed method is based on the naïve-Bayes approach that uses the sect of n-grams and the statistics calculated from the set of n-grams. The set of multi-n-grams can be enriched easily that provides fast CNER.

The extraction of CNEs is based on the prediction of each token to belong to the class CNE, the selection of tokens belonging to CNE based on the predefined threshold of probability values, and the further concatenation of selected tokens. We have applied our approach for a task of retrieving chemical named entities of potential inhibitors of SARS-CoV-2 Mpro as compounds that can slow down COVID-19 progression. As a result, we extracted 2749 unique chemical named entities and thoroughly evaluated the correctness of chemical named entity recognition. We thoroughly analysed extracted named entities, and for some of them, the experimental confirmation of their SARS-CoV-2 Mpro inhibition was found, and their role as anti-SARS-CoV-2/COVID-19 agents was discussed.

## Supplementary Information

**Additional file 1**. An SDFfile with the CHEMDNER corpus. Can be viewed using Knime or any SDFviewer. Is provided in gzip format.

**Additional file 2**. The set of text files extracted from NCBI PubMed and relevant to the inhibition of SARS-CoV-2 main protease (Mpro). A set of text files provided in gzip format.

**Additional file 3**. The IA, sensitivity (recall), precision, specificity, and BA values for various thresholds of B-statistics.

**Additional file 4**. The set of chemical named entities extracted from the texts relevant to the inhibition of SARS-CoV-2 (Mpro); some filters for CNE filtration are included. Is provided in MS Excel format.

**Additional file 5**. The SDF files created from CNEMDNER for 5-fold cross validation. A set of SDF files provided in gzip format. The results of naïve-Bayes CNER calculated for 5-fold cross validation using CHEMDNER, each file in CSV format. The results of CNER for the Sars-CoV-2 Mpro set.

## Data Availability

The proposed method of extracting CNEs from texts based on the naïve Bayes classifier is freely available as web service at http://way2drug.com/cner/. The recognition is performed for text fragments of three tokens (target token, one token before, and one token after target token, the so-called context [-1; 1]). The web-service produces the results of recognition presented as text fragments of three tokens, where target token is given in uppercase. We used the context [-1; 1], because for it the recognition accuracy is the highest among other context options. The Python 3.10 scripts that provide processing of the results of CNER results based on the naïve Bayes classifier are freely available on Github: https://github.com/olga-tarasova/CNER. Extraction-bayes-rests-compare.py is the script that includes processing of CNER results with automatic calculation of the number of true positives, false positives, true negatives and false negatives. It processes two files: the recognition results in CSV format, where tokens are saved explicitly, and the recognition results, where for each token its class is provided instead token itself. Collect-PubChem-results.py is the script that provides queries to the PubChem database and downloading data from this database. It returns the CNE and the label "1" if this CNE has been found in PubChem, and label "0", if it has not been found in the database. Chembl_request.py is the script providing Chembl requests. Extraction-bayes-rests.py is the script that provides the extraction of the tokens that belongs to CNEs in accordance with the recognition results and the formation of tokens' combinations for further analysis. http://way2drug.com/cner/
