# Supplementary material for: Chemical named entity recognition in the texts of scientific publications using the naïve Bayes classifier approach
Source: J Cheminform. 2022 Aug 13;14:55. doi: 10.1186/s13321-022-00633-4 (PMC9375066; doi:10.1186/s13321-022-00633-4)
Supplement: Supplementary file 2 — Additional file 2. The set of text files extracted from NCBI PubMed and relevant to the inhibition of SARS-CoV-2 main protease (Mpro). A set of text files provided in gzip format. [file 13321_2022_633_MOESM2_ESM.zip › Additional file 2/Scripts/collect-PubChem-results.py]

CNER/collect-PubChem-results.py at main · olga-tarasova/CNER · GitHub


Skip to content


Sign up

- Product

  - Features
  - Mobile
  - Actions
  - Codespaces
  - Copilot
  - Packages
  - Security
  - Code review
  - Issues
  - Integrations
  - GitHub Sponsors
  - Customer stories
- Team
- Enterprise
- Explore

  - Explore GitHub
  - Learn and contribute
  - Topics
  - Collections
  - Trending
  - Skills
  - GitHub Sponsors
  - Open source guides
  - Connect with others
  - The ReadME Project
  - Events
  - Community forum
  - GitHub Education
  - GitHub Stars program
- Marketplace
- Pricing

  - Plans
  - Compare plans
  - Contact Sales
  - Education

- In this repository

  All GitHub
  ↵

  Jump to
  ↵

- No suggested jump to results

- In this repository

  All GitHub
  ↵

  Jump to
  ↵
- In this user

  All GitHub
  ↵

  Jump to
  ↵
- In this repository

  All GitHub
  ↵

  Jump to
  ↵

Sign in

Sign up

{{ message }}

olga-tarasova
/
 **CNER** 
Public

- Notifications
- Fork
  0
- Star
   0

- Code
- Issues
  0
- Pull requests
  0
- Actions
- Projects
  0
- Wiki
- Security
- Insights

More

- Code
- Issues
- Pull requests
- Actions
- Projects
- Wiki
- Security
- Insights

Permalink

main

Switch branches/tags


Branches
Tags

Could not load branches


Nothing to show


{{ refName }}
default
View all branches

Could not load tags


Nothing to show


{{ refName }}
default

View all tags

## CNER/**collect-PubChem-results.py** / Jump to Code definitions Code navigation index up-to-date

 Go to file

 

- Go to file
  T
- Go to line
  L
- Go to definition
  R
- Copy path
- Copy permalink

This commit does not belong to any branch on this repository, and may belong to a fork outside of the repository.

olga-tarasova

Add files via upload

Latest commit
fe2e44c
Apr 20, 2022


**History**

**1**
contributor


### Users who have contributed to this file

43 lines (41 sloc)
1.48 KB

Raw
  Blame

Edit this file

E


Open in GitHub Desktop

- Open with Desktop
- View raw
- Copy raw contents
   Copy raw contents

   Copy raw contents

   Copy raw contents
- View blame

This file contains bidirectional Unicode text that may be interpreted or compiled differently than what appears below. To review, open the file in an editor that reveals hidden Unicode characters.
Learn more about bidirectional Unicode characters

Show hidden characters


|  |  |
| --- | --- |
|  | import os |
|  | import urllib.request as ull |
|  | import urllib as ul |
|  | import codecs |
|  | dirtory = 'D:\\Texts\\PASS-rests\\' |
|  | with codecs.open(dirtory + "out-chemlist-SARS-CoV-2.txt", encoding = "utf8") as f: |
|  | lines = f.readlines() |
|  | #lines = |
|  | i=1 |
|  | k=0 |
|  | n=0 |
|  | found = 1 |
|  | outlist = [] |
|  | while i < len(lines): |
|  | currsl = lines[i].split("\t") |
|  | crn = currsl[0] |
|  | crn = crn.replace('\\' , '-') |
|  | crn = crn.replace(' ' , '%20') |
|  | crn = crn.lower() |
|  | print (crn) |
|  | found = 1 |
|  | try: |
|  | ull.urlretrieve ('https://pubchem.ncbi.nlm.nih.gov/rest/pug/compound/name/' + crn + '/XML/', dirtory + "temp" + ".xml") |
|  | print("found") |
|  | # ull.urlretrieve ('https://pubchem.ncbi.nlm.nih.gov/rest/pug/compound/name/synonyms/hereisis/XML/', dirtory + "1-energy" + ".xml") |
|  | except ul.error.HTTPError: |
|  | print ("not found") |
|  | found = 0 |
|  | except UnicodeEncodeError: |
|  | print ("Encode Error") |
|  | found = 2 |
|  | #ull.urlretrieve ('https://www.ebi.ac.uk/chembl/api/data/similarity/CC1=CN([C@H]2C[C@H](N=[N+]=[N-])[C@@H](CO)O2)C(=O)NC1=O/100', dirtory + "1" + ".xml") |
|  | #ull.urlretrieve('https://eutils.ncbi.nlm.nih.gov/entrez/eutils/efetch.fcgi?db=pubmed&rettype=abstract&retmode=text&id=' + s, dirtory + s + ".txt") |
|  | #ull.urlretrieve ('https://eutils.ncbi.nlm.nih.gov/entrez/eutils/efetch.fcgi?db=pubmed&rettype=abstract&retmode=xml&id=' + s, dirtory + s + ".xml") |
|  | #k=0 |
|  | outlist.append(str(found)+ " " + lines[i]) |
|  | i=i+1 |
|  |  |
|  | file = open(dirtory + "outpubchemlist\_SARS-CoV-2-drugs.txt", 'w') |
|  | file.writelines(outlist) |
|  | file.close() |
|  |  |
|  | # |

- Copy lines
- Copy permalink
- View git blame
- Reference in new issue

 Go

- © 2022 GitHub, Inc.

- Terms
- Privacy
- Security
- Status
- Docs
- Contact GitHub
- Pricing
- API
- Training
- Blog
- About

You can’t perform that action at this time.

You signed in with another tab or window. Reload to refresh your session.
You signed out in another tab or window. Reload to refresh your session.
